# Oral Medicine and Oral Clinical Chemistry Game Changers for Future Plaque Control and Maintenance: PerioSafe^®^ aMMP-8 POCT, Lumoral^®^ 2× PDT- and Lingora^®^ Fermented Lingonberry Oral Rinse-Treatments

**DOI:** 10.3390/dj13030127

**Published:** 2025-03-13

**Authors:** Nur Rahman Ahmad Seno Aji, Vaibhav Sahni, Miika T. Penttala, Dimitra Sakellari, Andreas Grigoriadis, Tommi Pätilä, Pirjo Pärnänen, Dirk Neefs, Andreas Pfützner, Shipra Gupta, Timo Sorsa, Ismo T. Räisänen

**Affiliations:** 1 Department of Oral and Maxillofacial Diseases, Head and Neck Center, University of Helsinki and Helsinki University Hospital, 00290 Helsinki, Finland; 2 Department of Periodontics, Faculty of Dentistry, Universitas Gadjah Mada, Jalan Denta No. 1 Sekip Utara, 10 Sleman, Yogyakarta 55281, Indonesia; 3 Research & Evidence (RF&E), New Delhi 110001, India; 4 Department of Preventive Dentistry, Periodontology and Implant Biology, Dental School, Aristotle University of Thessaloniki, 54124 Thessaloniki, Greece; 5 Dental Sector, 424 General Military Training Hospital, 56429 Thessaloniki, Greece; 6 Department of Congenital Heart Surgery and Organ Transplantation, New Children’s Hospital, University of Helsinki, 00290 Helsinki, Finland; 7 Department of Biomedical Surgical and Dental Sciences, Faculty of Medicine and Surgery, University of Milan, 20122 Milan, Italy; 8 Department of Internal Medicine and Laboratory Medicine, University for Digital Technologies in Medicine and Dentistry, 9516 Wiltz, Luxembourg; 9 Oral Health Sciences Centre, Post Graduate Institute of Medical Education & Research, Chandigarh 160012, India; 10 Division of Oral Diseases, Department of Dental Medicine, Karolinska Institutet, 17177 Stockholm, Sweden

**Keywords:** aMMP-8 POCT biomarker, 2× PDT light treatment, fermented lingonberry juice oral rinse, oral clinical chemistry

## Abstract

**Background**: Periodontitis is a global health crisis that affects almost half of the world’s population and commonly goes unnoticed because of its asymptomatic and pain-free nature. For early and easy detection and treatment, safe and non-invasive chair-side oral fluid biomarker (aMMP-8) diagnostics and new anti-microbial, anti-inflammatory and anti-proteolytic treatment modalities have been developed, which this review aims to introduce. **Methods**: For convenient diagnosis and tackling of periodontitis, adoption of an oral fluid aMMP-8 chair-side point-of-care rapid diagnostic test (POCT) has been proposed, comparable to home pregnancy and COVID-19 antigen tests, to be conveniently used by healthcare professionals and by patients themselves. To improve treatment of detected periodontitis, Finnish scientists have also developed a potentially industry-altering, biofilm-modulating, anti-microbial, anti-inflammatory, and anti-proteolytic (i) dual-light-activated photodynamic-therapy (2×PDT) and (ii) fermented lingonberry juice (FLJ) oral rinse designed for home personalized medicine and professional use. These new oral medicine technologies are reviewed and some unpublished results are presented. **Results**: aMMP-8 is the superior biomarker for grade of periodontitis (progression rate) when compared to the total latent/proform MMP-8 (total-MMP-8) and microbial lipopolysaccharide (LPS/LAL) activity. Cut-off 20 ng/mL is the optimal cut-off for aMMP-8 POCT and does not make false positives. Antibacterial 2× PDT light and anti-microbial FLJ treatments can eliminate and reduce problem-causing bacteria and Candida-yeasts from the mouth. **Conclusions**: These new oral medicine technologies have shown promising results and could have the potential to revolutionize diagnosis, prevention, oral care, plaque control and maintenance. These new game-changer oral medicine technologies have launched a new clinical field in dentistry: oral clinical chemistry.

## 1. Introduction

Periodontitis is an inflammatory infectious disease that causes the breakdown of the collagen fibers that attach the teeth to the jawbone. In severe and advanced cases of periodontitis, teeth may loosen or even detach entirely. Early signs of periodontitis include red gums, bleeding gums, accumulated dental plaque and bad breath [1,2,3,4].

Globally, more than 700 million people have a severe form of periodontitis and, in western countries, more than 10% of the population has severe periodontitis [3]. In such cases, the inflamed area around the tissues supporting teeth can be as large as the palm of a hand in terms of its surface area. Even in milder cases, there is an inflamed ‘wound surface’ causing low-grade systemic inflammation in the body [1,2].

Untreated periodontitis, which impacts the tissues that support the tooth, elevates the body’s chronic low-grade systemic inflammation, linking it to systemic chronic diseases, like diabetes, cardiovascular diseases, Alzheimer’s disease, rheumatoid arthritis, and even pancreatic cancer [1,2,3,4,5,6]. Low-grade systemic inflammation is a condition where the body responds to persistent irritation by the release of various disease promoting pro-inflammatory mediators and proteolytic enzymes [6]. The sustained inflammation thus caused, while considered ‘mild’, can nonetheless become harmful to the body over time. Individuals with periodontitis may even have a higher risk of developing serious forms of cancer [7]. According to a recent study [7], individuals aged 50–70 with periodontitis had a 20% higher likelihood of developing pancreatic cancer compared to participants with healthy gums. Furthermore, the risk of developing cancer was also elevated in patients who had already lost several teeth, indicating experience of advanced periodontitis. The study utilized registry data encompassing nearly six million individuals aged 19 and above residing in Sweden [7].

## 2. Hidden Periodontitis Uncovered?

With such grave consequences in mind, the early identification and treatment of periodontitis assumes greater significance. Initial or early periodontitis is far easier to treat than advanced periodontitis [1,2,3,4].

Given the association between periodontitis and various systemic diseases, the timely detection and treatment of this condition are of the greatest importance for overall systemic well-being and quality of life. It is also equally important to have detection methodology in place which is not overly complex and provides results in a timely manner with adequate sensitivity and specificity [8]. In this regard, Finnish researchers have now developed an effective method to address this critical aspect [9]. The active matrix metalloproteinase-8 (aMMP-8) chair-side oral fluid lateral flow (LF) immunotest has been independently and repeatedly confirmed by others [10,11,12,13,14,15]. Matrix metalloproteinase-8 or collagenase-2 (also called neutrophil collagenase) is primarily a proteolytic enzyme produced and degranulated by human white blood cells, or neutrophils, and its role is to enable the passage of collagenolytic neutrophils to the inflamed area by modifying the extracellular matrix and immune responses [16,17]. Latent degranulated non-collagenolytic and non-proteolytic catalytically non-competent and enzymatically inactive proMMP-8 (total MMP-8) can be activated to collagenolytic and proteolytic catalytically competent and enzymatically active MMP-8 (aMMP-8) by independent and/or co-operative action of other MMPs, serine proteases, bacterial and Candida proteases, as well as by reactive oxygen species [16,17].

In patients with periodontitis, the latent proMMP-8 enzyme thus becomes catalytically active MMP-8 (aMMP-8) and proteolytically and collagenolytically tissue-destructive due to its abundant presence and destructive action [8,10,11,12,13]. The LF aMMP-8 chairside point-of-care test (aMMP-8 POCT) can measure and assess active and progressive collagenolytic periodontal and peri-implant attachment loss due to aMMP-8 within five minutes directly in a point-of-care test (POCT) at the dentist’s chair, by a non-invasive procedure, i.e., without invasive tissue examination and bacteremia [8,9,10,11,12,13,14,15,16,17,18,19,20,21,22]. The LF aMMP-8 POC immunotest complements the traditional diagnostics of periodontitis and peri-implantitis, monitors online and in real-time and reflects treatment outcomes, and supports maintenance therapy [8,9,10,11,12,13,14,15,16,17,18,19,20,21,22].

Two LF aMMP-8 PoC chair-side test kits have been developed—PerioSafe^®^ and ImplantSafe^®^—to analyze the levels of active collagenolytic aMMP-8—but not total, latent non-collagenolytic proMMP-8—in oral fluids (mouth rinse, saliva, gingival crevicular fluid [GCF] and peri-implant sulcular fluid [PISF]). The LF immunokits are like a COVID-19 antigen test or a classical pregnancy test, providing a visual result of two lines when positive, indicating a higher risk of periodontitis/peri-implantitis. These tests are inexpensive, noninvasive, do not require specialized equipment or trained staff and provide a quick online, real-time outcome [8,17,23].

Once the presence of active MMP-8 has been identified in the oral fluid sample, a quantitative analysis can be performed using the ORALyzer^®^, a commercially available quantitative reader-based technology [13,22], to assess aMMP-8 levels in the LF immunotests. The results can thus be both qualitative (visual +/−) and quantitative via the use of the ORALyzer reader, with a cut-off at 20 ng/mL in 5–7 min [13].

These tests with a cut-off of 20 ng/mL have been successfully clinically and independently validated in Finland, Nigeria, Germany, Holland, Mali, Türkiye, India, Italy, Chile, Sweden, and USA. The tests have diagnostic sensitivity and specificity of 76–90% and 96%, respectively, corresponding to an odds ratio of >72 [8,18,21,24]. The tests with a cut-off of 20 ng/mL have rarely or hardly ever been observed to make false positive outcomes so far and, hence, can be considered as test technologies with high precision [8,13,16,24]. The LF aMMP-8 PoC test results can thus be made quantitatively available at the chair-side by the reader in five minutes. The tests have demonstrated utility in screening susceptible sites and patients, differentiating active and inactive periodontitis and peri-implantitis sites and patients, predicting future disease progression, and monitoring treatment outcomes and assisting maintenance [8,13,24]. The tests are able to detect and warn of periodontitis in its initial stages, thereby making visible what is invisible, prior to clinical and radiographic manifestations [8,13,17,23,24]. The screening can be performed in the absence of trained dental professionals, by medical professionals or even by the patients themselves [8,13,17,23,24]. Patients tested positive are referred to oral health professionals (dentists and/or oral hygienists) for the required therapeutic interventions [8,13,24].

The PerioSafe^®^ and ImplantSafe^®^ LF aMMP-8-POCT kits are efficient and handy tools for improvement of diagnostic and prognostic accuracy for periodontal or peri-implant diseases, and are commercially available and approved technologies by the FDA/USA and EU. PerioSafe^®^, ImplantSafe^®^ and ORALyzer^®^ technologies have been validated as functioning as a single biomarker, aMMP-8, that has been successfully implemented by Sorsa et al. with a cut-off of 20 ng/mL as the requisite biomarker of significance in the new staging and grading classifications for both periodontitis and peri-implantitis [4,8,16,17,18,24]. aMMP-8 POCT is available as both mouthrinse and peri-implant sulcular fluid/gingival crevicular fluid (PISF, GCF) variants [24]. The difference between both tests is that PerioSafe^®^ indicates the general periodontal status at patient level, whereas the ImplantSafe^®^ variant can be used as a site-specific GCF or PISF test [8,24].

The aMMP-8 POCT with Oralyzer has recently been repeatedly demonstrated to be the most precise biomarker in discriminating periodontal health and disease [8,24]. Second in terms of precision is the aMMP-8 catalytic activity assay (not immunolevels) analysed by an independent catalytic aMMP-8 RFU-activity assay [8,25]. The aMMP-8 POCT and aMMP-8 catalytic RFU-activity assays complement each other and correlate well with each other [8,25]. These independent aMMP-8 assay technologies are superior to total MMP-8 ELISA [8,25]. This is apparent from the ROC analyses of these tools, wherein there is a clear superiority in terms of sensitivity, specificity, and precision for the aMMP-8 analysis vs. total MMP-8 analysis [8,25].

aMMP-8 has also been evidenced to be superior in discerning between grades of periodontitis [9,14,26]. When comparing 150 Greek adult patients, aMMP-8 levels were statistically significantly associated with grade of periodontitis and its levels were observed to be statistically significant in pairwise differences between grades A and B and grades A and C, as has been previously reported [9]. This suggests that low aMMP-8 levels are associated with lower grades of periodontitis. Similarly, statistically significant associations have been previously observed with stage of periodontitis when aMMP-8 was factored in as a function of the number of teeth present (aMMP-8/NTP) [14,26]. Furthermore, there was a significant association between aMMP-8/NTP and grade of periodontitis among 150 Greek adults with periodontitis (Table 1). In contrast, the total MMP-8 biomarker [26], which is detecting both inactive pro-/latent- and active species of MMP-8), had much larger variability in its levels than collagenolytic aMMP-8 and was not statistically significantly associated with grade of periodontitis among 150 Greek adult patients (Table 1).

It seems that the large variability in total MMP-8 levels may be due to the levels of pro-/latent species of MMP-8 among Greek periodontitis patients, and thus total MMP-8 did not show statistically significant differences between the various grades of periodontitis, while aMMP-8 levels did reach the level of significance (Table 1). The aMMP-8 and total MMP-8 results are in agreement with previous findings, e.g., Lee et al. and Romanelli et al. [10,11], that it is the collagenolytic and proteolytically active aMMP-8, and not the inactive pro-/latent species of MMP-8, that plays the more important role in the pathological periodontal tissue breakdown and progression of periodontitis.

Finally, there was no statistically significant association between bacterial lipopolysaccharide (LPS) activity and grade of periodontitis, and no differences were found for LPS activity (Table 1), as evaluated by the LPS(LAL)-activity [27], in differentiating between the different grades of periodontitis. In that regard, it should be noted that previously, in the same sample of 150 Greek adults, it was reported that the visible dental plaque index showed statistically significant association with grade of periodontitis [9]. These findings underscore the value of maintaining good oral hygiene levels (regardless of LPS(LAL)-activity) for patients with periodontitis in order to control bacterial growth and prevent/slow down the progression of their disease.

## 3. A Tool Against Systemic Diseases

The aMMP-8 test measures the activity of periodontal and peri-implant diseases in order to differentiate between diseased and healthy tissues with good accuracy [8,12,20,21,23]. In patient care, a negative result in the aMMP-8 test is aimed for, which indicates healthy tissue with a low risk of attachment loss progression or clinical disease progression. Low, safe ([−], <20 ng/mL) levels can also be regarded as an indicator and a biomarker of periodontal or peri-implant health [8,24].

**Figure 1:**
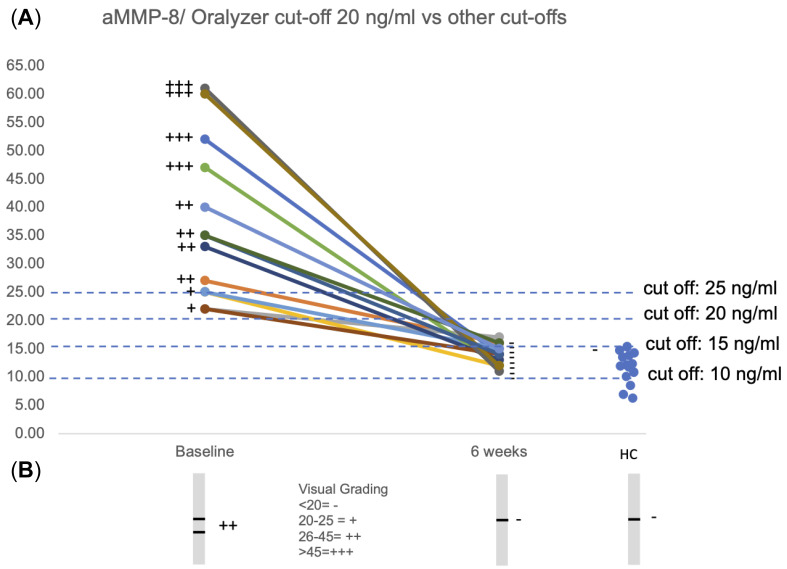
(**A**,**B**) Monitoring of the anti-infective scaling and root planing treatment of ten Finnish chronic periodontitis stage III/IV grade b/c adult patients by aMMP-8 POC quantitated by Oralyzer (**A**) and visually (**B**), as described previously regarding patients and biomarker analysis methods [13]. Baseline (before treatment) and 6 weeks after clinically successful treatment are shown. Thirteen Finnish periodontally and systematically healthy 3rd year dental students served as healthy controls (HCs). Visual grading is indicated as (+++, ++, +) representing positive tests and (-) representing negative tests. Different cut-offs 10, 15, 20, and 25 ng/mL were compared. Observational study.

This is evident from Figure 1, which represents visual grading data for patients, which clearly demarcates 20 ng/mL as the optimal cut-off point to differentiate between healthy controls and those with periodontitis in a Finnish cohort (Figure 1A,B). This has also been observed to be true across a variety of cohorts, including Turkish, Indian, Greek, Italian, Nigerian, Mali, Chinese, US, and Swedish populations [8,9,13,16,18,24,28,29,30,31]. The effect of anti-infective scaling and root planing treatment performed for stage III/IV grade b/c-periodontitis patients was monitored as described previously [11] by visual and Oralyzer quantitated aMMP-8 POCT, and the data between (t0, baseline [BL]) after (t1, 6 weeks) shows clinically effective anti-infective treatment, as seen in Figure 1A,B. Upon analysis of cut-offs at 10 ng/mL, 20 ng/mL and 25 ng/mL, the 20 ng/mL mark was found to be optimal. Thirteen systemically and periodontally healthy 3rd year dental students served as healthy controls (Figure 1A). As has been observed in an earlier study, the 10 ng/mL cut-off point [14,15,16,17] is not reliable in the clinical assessment and monitoring of patients undergoing periodontal therapy scaling and root planing and anti-infective treatment [13]. All periodontitis patients’ pre-treatment aMMP-8 levels were over 20 ng/mL and their post-treatment aMMP-8 levels below 20 ng/mL, but not below 10 ng/mL, after successful treatment (Figure 1A). Thus, periodontitis patients earlier reported to be aMMP-8 positive with cut-off at 20 ng/mL were deemed negative upon utilization of the 10 ng/mL cut-off point. On the other hand, all healthy controls (13 systemically and periodontally healthy) had aMMP-8 POCT results visually (-) and ≤20 ng/mL, indicating periodontal health (Figure 1A,B). However, most did not have aMMP-8 levels below 10 ng/mL. Thus, visually negative (-) and ≤20 ng/mL aMMP-8 was the optimal cut-off, making no false positives, and can be regarded as the biomarker of periodontal health (Figure 1A,B).

The current oral health diagnostic and treatment protocols could be significantly enhanced by the regular incorporation of the aMMP-8 rapid test. This is particularly important, as periodontitis and diabetes have been observed to have an interlinked bidirectional relationship [32,33]. In fact, the aMMP-8 POCT has been demonstrated to correlate positively with glycated HbA1c [23]. Previous studies have found increased aMMP-8 levels in gingival extracts of periodontitis patients, where diabetes further elevates aMMP-8 levels compared to non-diabetics [34]. Thus, aMMP-8 testing could conveniently aid in identifying and alarming not only periodontitis but also hidden diabetes or diabetic disease development.

The aMMP-8 test can be used not only by individuals at home but also in medical clinics for periodontitis screening, helping to screen and identify patients who need to be referred to the dentist or oral hygienist for clinical examination and treatment of gum disease [24]. In this regard, aMMP-8 POCT represents the “optimal” periodontitis single biomarker test, being capable of providing, predictively and accurately, the on-line, real-time and chair-side diagnosis and screening of periodontitis in 5 min in the absence of a periodontist or dentist [8,17,24]. aMMP-8 POCT has been found to be useful in identifying periodontitis as an escalating risk disease during COVID-19 infections [35].

The aMMP-8 enzyme test can also accelerate and confirm the diagnosis of gum diseases in implant patients before implant surgery. It would be advisable not to insert an implant into the mouth with teeth that show positive ([+], >20 ng/mL) for the aMMP-8 POCT PerioSafe^®^ [21,22] test. aMMP-8 especially, rather than total MMP-8, is related to and reflects clinically progressive periodontitis and peri-implantitis [8,21,26], and aMMP-8 is thus not synonymous with total MMP-8 in periodontitis and peri-implantitis diagnostics. Regarding oral fluids as matrices to biomarkers, oral mouthrinse technologies are more precise than saliva biomarker analyses in respect to aMMP-8, -9, and neutrophil elastase [16,36,37]. While traditional methods involve culturing potential bacterial pathobionts in laboratories, which takes days to weeks [38], the aMMP-8 POCT as a single biomarker provides a quicker (chair-side in 5 min) and more precise alternative for identifying and handling periodontal issues in patients before implant placement or other medical procedures [8,24].

It is noteworthy that diabetes and its treatment outcomes can be positively affected by efficient and prompt diagnostics, screening, and treatment of periodontitis, which can also potentially lead to a reduction in treatment costs [32,33]. Conversely, successful screening and treatment of diabetes not only slows down the progression of periodontitis but also plays a role in decreasing associated treatment costs [32,33].

## 4. Bacteria Spread Through the Gums

In addition to rapid diagnosis, there is an urgent need for more effective methods to eliminate problem-causing dysbiotic bacteria, i.e., dental plaque accumulation and its dysbiosis from the oral cavity. In periodontal health, there is a homeostatic state, a healthy balance, between the host immune response and subgingival biofilm [2]. However, this homeostasis can be disrupted in a susceptible individual in the interplay between inflammation and the conversion of the polymicrobial community from the original homeostatic community into a dysbiotic community [39]. It is known that dysbiosis can promote destructive inflammation, while inflammation can promote development of a nutritional environment (with tissue breakdown products, such as degraded collagen) that favors the selection of pathobiotic bacteria (dysbiosis) [2]. This forms a feed-forward loop, which can eventually lead to development of periodontitis [2].

Research indicates that 95% of all oral diseases are attributed to uncontrolled plaque accumulation. The harmful bacteria present in plaque can escalate the risk of severe conditions, like diabetes and Alzheimer’s disease, and escalate respiratory and COVID-19 infection, as well as cancers [5,6,35]. Oral dysbiotic bacteria can thus be found in periodontobionts’ biofilms, and their disseminated virulence factors can promote the development and affect the course of serious types of cancer [7].

**Figure 2:**
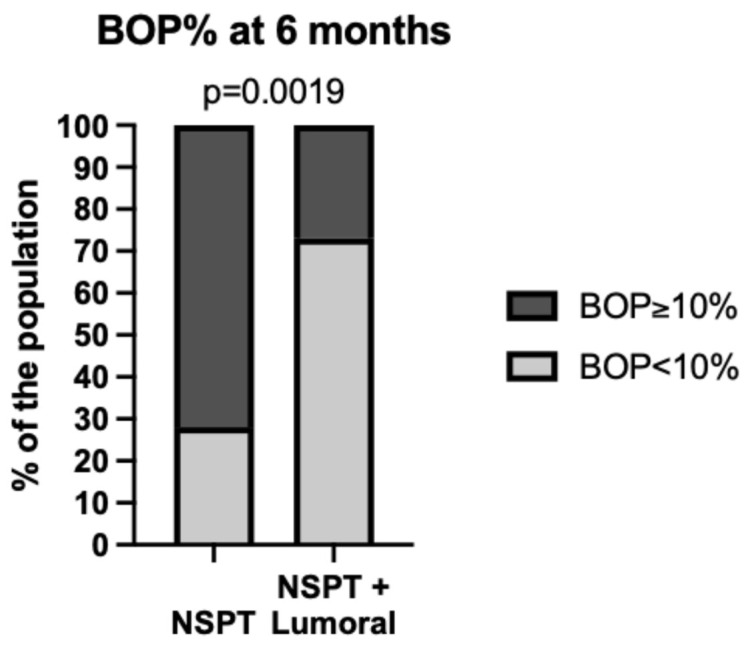
Lumoral treatment as an adjunct improves the results of gold standard non-surgical periodontal treatment (NSPT)/anti-infective treatment. Significantly more patients reach the target BOP level of less than 10% (27 Finnish adult participants in the NSPT group and 24 adult participants in the NSPT + Lumoral group all having stage I–III periodontitis, as described in detail in Pakarinen et al. [40]). Observational study.

Merely brushing one’s teeth is insufficient to eliminate these harmful bacteria, as research has shown. Mechanical brushing is a crucial step that removes about 65% of the accumulated plaque, but there is an absolute need to continue doing this regularly. In order to rid the mouth completely of harmful and dysbiotic bacteria, a complement is required. For this purpose, Finnish researchers have developed an antibacterial 2× PDT Lumoral^®^-light treatment device and anti-microbial Lingora^®^ fermented lingonberry juice oral rinse treatment that target the dysbiotic oral microbiome and eliminate and reduce problem-causing bacteria and Candida-yeasts from the mouth [40,41,42,43]. These technologies are also designed for home use. They serve as complementary tools to conventional oral hygiene practices, like brushing and flossing, and are complementary to each other [40,41,42,43]. They can be regarded as enhancers of tooth-brushing and as oral hygiene and maintenance promoters. In addition, 2× PDT Lumoral^®^ light treatment as an adjunct to non-surgical periodontal treatment (NSPT) or anti-infective treatment of periodontitis improved target BOP levels to less than 10% among Finnish stage I–III periodontitis patients (Figure 2) [40].

**Figure 3:**
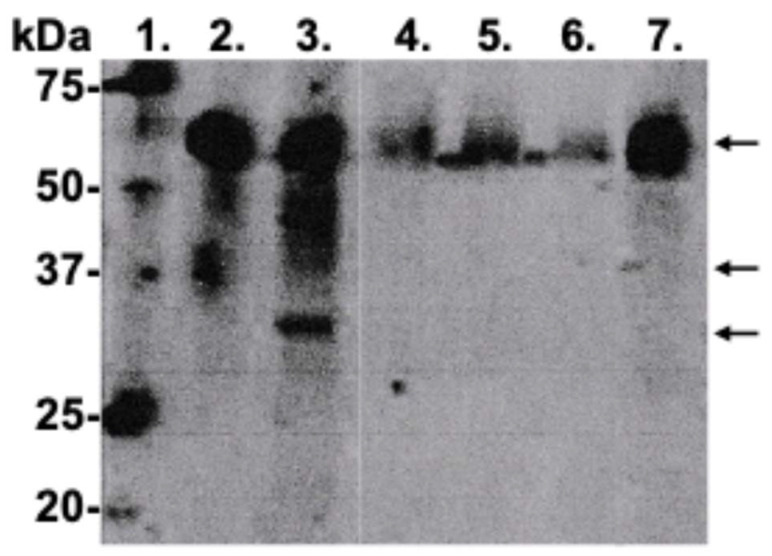
Activation of ProMMP-8 by *Candida glabrata* protease [43] and its inhibition by Lingora^®^. Lane 1: molecular weight standard. Lane 2: proMMP-8 [0.8 μg] treated with 1.0 mM APMA 3 h. Lane 3: proMMP-8 [0.8 μg] treated with *Candida glabrata* protease [5 μL] overnight. Lane 4: as lane 3 plus Lingora^®^ pretreatment 5 μL overnight. Lane 5: as lane 3 plus Lingora^®^ pretreatment 10 μL overnight. Lane 6: as lane 3 plus Lingora^®^ pretreatment 20 μL overnight. Lane 7: ProMMP-8 [0.8 μg] treated with TNC buffer 3 h (on the right the arrows indicate proMMP-8, active MMP-8, activation-related lower molecular size MMP-8 fragments). Lingora^®^ 1:1 dilution, Pärnänen, P.; EP2 585 087B1, study described in detail in [43]. The mobilities of the molecular weight markers are indicated. Observational study.

**Figure 4:**
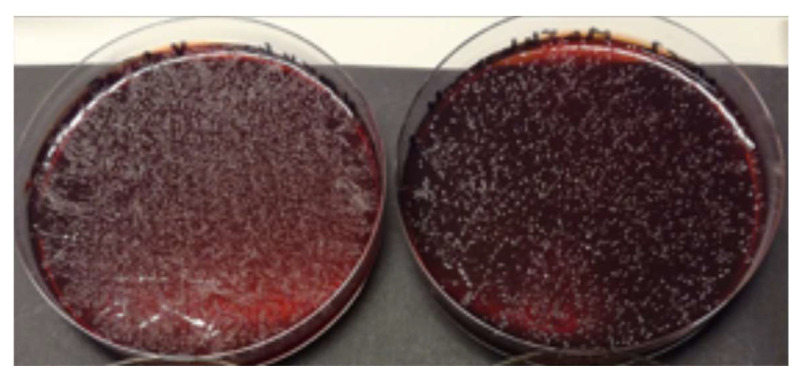
Cultivation of obligate anaerobe microbes before and after Lingora^®^ oral rinse treatment. 100 µL of oral saline rinse cultivated on Brucella Agar: left plate 0 d, right plate after 14 d use of Lingora^®^ oral rinse (10 mL × 2 for 30 s) (study described in [42]). 10^−1^ dilutions are shown, corresponding to 1 × 10^7^ cfu/mL (0 d) and 1 × 10^5^ cfu/mL (14 d) oral undiluted colony counts. Observational study.

Being anti-proteolytic and anti-inflammatory in their action, Lumoral^®^ 2× PDT-light treatment and Lingora^®^ oral rinse can also reduce the elevated (>20 ng/mL) aMMP-8 levels in mouthrinse, GCF and PISF, close to those observed in healthy controls [40,41,42,43]. Lumoral^®^ can produce an excessive amount or “storm” of reactive oxygen species that attack the dental biofilm and, with this antibacterial effect, can eventually inactivate or inhibit MMP-8 activation to aMMP-8 [40]. Lingora^®^ oral rinse has been shown to be capable of inhibiting latent proMMP-8 activation by the *Candida* protease to aMMP-8 (Figure 3), as well as inhibiting growth of obligate anaerobe microbes (Figure 4) [42,43]. Samples for evaluating the effect of Lingora^®^ on oral obligate anaerobe bacteria were collected by rinsing 10 mL 0.9% NaCl for 30 s. Cultivation and colony counting were performed by serial dilution (Figure 4). Moreover, the Western blot results in Figure 3 represent the new microbial mechanism presented, i.e., a candidal activation of 75–80 kD latent proMMP-8 to lower molecular size of active MMP-8 and related lower molecular size fragments (Figure 3, lane 3) (similar to MMP-8 activation by APMA, Figure 3, lane 2). Pretreatment of the *Candida glabrata* protease using Lingora^®^ prevented this (Figure 3, lanes 4–6), with the outcome that the 75–80 kD latent proMMP-8 remained unconverted/untouched, as indicated by arrows on the right. Furthermore, 75–80 kDa latent proMMP-8 remains unconverted with TNC-buffer (Figure 3, lane 7). The data in Figure 3 confirm and further extend the experiment by Pärnänen et al. [42,43].

## 5. How Can One Prevent Dysbiotic Bacterial Buildup?

Brushing one’s teeth thoroughly and using dental floss or interdental brushes is the first step. One also needs to visit the dentist or dental hygienist regularly. Adding regular antibacterial light 2× PDT-treatment and Lingora^®^ oral rinse to the oral hygiene routine is useful for everyone but is particularly important for those who have already been affected with tooth loss or gum inflammation or systematically affected. The 2× PDT-lumoral^®^ light and anti-microbial Lingora^®^oral rinse treatments hamper plaque formation and significantly reduces the number of dysbiotic periodontal bacteria and Candida, as well as reducing aMMP-8 in the mouthrinse GCF and PISF [40,41,42,43]. Elderly people, for example, are particularly well suited to the user profile presented by this methodology, as optimal oral self-care is critical in this age group.

Initial observations suggest that antibacterial 2×PDT Lumoral light therapy might even be an important tool in the armory for oral mucositis treatment in head and neck cancer patients undergoing treatment, particularly radiation and chemotherapy. The clinical progression of periodontitis, measurable as increases in CAL, VPI, BOP and related oral mucositis due to head and neck cancer radiotherapy, can be predictively and quantitatively diagnosed in a chair-side and real-time manner, as well as with screening by the aMMP-8 POCT [24,27]. At the same time, this test reveals the whole pro-collagenolytic, pro-gelatinolytic (MMP-9), pro-oxidative (MPO) and proinflammatory (IL-6) tissue destruction cascade accompanying aMMP-8 POCT [8,24]. This whole collagenolytic and pro-oxidative tissue destructive cascade in oral fluids can thus be identified by aMMP-8 POCT in 5 min, not only in diabetic disease or COVID-19 infection developments but also in head and neck cancer patient’s oral mucositis-related progressive periodontitis development [23,34,35]. Thus far, no existing medication has effectively prevented the development of cancer radiotherapy-induced mucositis and periodontitis. However, a recent study indicated that antibacterial 2×PDT Lumoral^®^ light therapy may be an effective method for management of the symptoms of this cancer radiotherapy-induced severe oral condition [40,41]. These patients will be tested also for therapeutic intervention with Lingora^®^ oral rinse treatment [42].

## 6. Conclusions

Periodontitis, a global health problem affecting almost half of the world’s population, commonly goes unnoticed because of its asymptomatic and pain-free nature. For easy and early detection and tackling of periodontitis, the adoption of an oral fluid chair-side point-of-care (POC) aMMP-8 rapid diagnostic test (aMMP-8 POCT, PerioSafe^®^, ImplantSafe^®^) has been proposed, comparable to home pregnancy and COVID-19 antigen tests, that can conveniently be used by healthcare professionals and by patients themselves. Furthermore, to improve the treatment of detected periodontitis, Finnish scientists have also developed a potentially industry-altering, biofilm modulating, anti-bacterial, anti-inflammatory, and anti-proteolytic (i) dual light-activated photodynamic-therapy (2×PDT, Lumoral^®^) and (ii) Fermented lingonberry juice oral rinse (Lingora^®^), designed for both professional and home personalized medicine use. These are safe and non-invasive without harmful side-effects, enhancing both tooth brushing and oral hygiene and maintenance. These oral medicine technologies have shown promising results in previous studies and have the potential to revolutionize diagnostics, prevention, oral care, plaque control and maintenance. However, more studies in large enough populations are recommended and needed to confirm previous promising findings and to further increase our knowledge. These new game-changer oral medicine technologies have launched a new clinical field in dentistry: oral clinical chemistry.

## Figures and Tables

**Table 1:** Biomarker levels of mouthrinse aMMP-8 and aMMP-8/NTP, total MMP-8, HbA1c, and LPS/LAL bacterial endotoxin activity assay according to 150 Greek adults, as described previously regarding patients and biomarker analysis methods [9,26,27]) and in relation to their periodontitis grade (risk of progression). Observational study.

	Grade A (N = 14) (Median ± IQR, 95% CI, [min, max])	Grade B (N = 91) (Median ± IQR, 95% CI, [min, max])	Grade C (N = 14) (Median ± IQR, 95% CI, [min, max])	*p*-Value	Significant Pairwise Differences
aMMP-8 (ng/mL)	15.0 ± 10.0, 5.0–15.0, [5.0, 20.1]	15.0 ± 18.9, 15.0–23.4, [5.0, 86.7]	28.3 ± 29.1, 15.0–53.5, [10.2, 73.1]	<0.001	Between grade A and B, grade A and C
aMMP-8 (ng/mL)/NTP	0.59 ± 0.51, 0.21–0.75, [0.18, 0.91]	0.77 ± 0.70, 0.63–0.90, [0.18, 4.3]	1.30 ± 1.30, 0.58–2.3, [0.46, 4.9]	0.002	Between grade A and B, grade A and C
Total MMP-8 (ng/mL)	38.8 ± 37.5, 22.9–62.4, [7.3, 136.4]	39.4 ± 74.8, 26.2–61.1, [0.22, 685.9]	60.2 ± 140.5, 24.3–174.7, [3.8, 289.3]	0.499	-
HbA1c (%)	5.35 ± 0.65, 4.8–5.6, [4.5, 6.2]	5.30 ± 0.60, 5.1–5.4, [4.1, 8.1]	5.95 ± 1.35, 5.4–6.9, [5.0, 8.9]	0.001	Between grade A and C, grade B and C
LPS-activity, LAL (EU/mL)	2760.5 ± 4095.0, 257.9–5531.8, [164.5, 6710.6]	1609.3 ± 3336.1, 1032.9–2582.1, [154.2, 7966.0]	991.2 ± 4147.7, 230.3–4781.8, [193.7, 7308.9]	0.683	-

NTP: Number of teeth present. IQR: Interquartile range. CI: confidence interval of median value. LAL: Lipopolysaccharide (LPS) activity measurement by LAL bacterial endotoxin assay (EU/mL). *p*-values calculated by Kruskal–Wallis test, post-hoc testing by Dunn–Bonferroni test.

## Data Availability

The data supporting the findings of this study are available upon reasonable request from the corresponding author. The data are not publicly available due to privacy and ethical restrictions.
